# Mcl-1 confers protection of Her2-positive breast cancer cells to hypoxia: therapeutic implications

**DOI:** 10.1186/s13058-016-0686-4

**Published:** 2016-02-26

**Authors:** Muhammad Hasan Bashari, Fengjuan Fan, Sonia Vallet, Martin Sattler, Melissa Arn, Claudia Luckner-Minden, Henning Schulze-Bergkamen, Inka Zörnig, Frederik Marme, Andreas Schneeweiss, Michael H. Cardone, Joseph T. Opferman, Dirk Jäger, Klaus Podar

**Affiliations:** Department of Medical Oncology, National Center for Tumor Diseases (NCT), University of Heidelberg, Im Neuenheimer Feld #460, Heidelberg, 69120 Germany; Department of Pharmacology and Therapy, Faculty of Medicine, Universitas Padjadjaran, Jl. Eijkman 38, Bandung, 02215 Indonesia; Dana-Farber Cancer Institute, 450 Brookline Avenue, Boston, MA 02215 USA; Eutropics, Inc., 767C Concord Avenue, Cambridge, MA 02138 USA; German Cancer Research Center (DKFZ), Im Neuenheimer Feld 460, 69120 Heidelberg, Germany; St. Jude Children’s Research Hospital, 262 Danny Thomas Place, Memphis, TN 38105 USA

**Keywords:** Breast cancer, Myeloid cell leukemia-1, Hypoxia, Apoptosis

## Abstract

**Background:**

Molecular mechanisms leading to the adaptation of breast cancer (BC) cells to hypoxia are largely unknown. The anti-apoptotic Bcl-2 family member myeloid cell leukemia-1 (Mcl-1) is frequently amplified in BC; and elevated Mcl-1 levels have been correlated with poor prognosis. Here we investigated the pathophysiologic role of Mcl-1 in Her2-positive BC cells under hypoxic conditions.

**Methods:**

RNA interference and a novel small molecule inhibitor, EU-5346, were used to examine the role of Mcl-1 in Her2-positive BC cell lines and primary BC cells (sensitive or intrinsically resistant to Her2 inhibitors) under hypoxic conditions (using a hypoxic incubation chamber). Mechanisms-of-action were investigated by RT-PCR, mitochondrial isolation, as well as immunoprecipitation/blotting analysis, and microscopy. The specificity against Mcl-1 of the novel small molecule inhibitor EU5346 was verified in Mcl-1^Δ/null^*versus* Mcl-1^wt/wt^ Murine Embryonic Fibroblasts (MEFs). Proliferation, survival, and spheroid formation were assessed in response to Mcl-1 and Her2 inhibition.

**Results:**

We demonstrate for a strong correlation between high Mcl-1 protein levels and hypoxia, predominantly in Her2-positive BC cells. Surprisingly, genetic depletion of Mcl-1 decreased Her2 and Hif-1α levels followed by inhibition of BC cell survival. In contrast, Mcl-1 protein levels were not downregulated after genetic depletion of Her2 indicating a regulatory role of Mcl-1 upstream of Her2. Indeed, Mcl-1 and Her2 co-localize within the mitochondrial fraction and form a Mcl-1/Her2- protein complex. Similar to genetically targeting Mcl-1 the novel small molecule Mcl-1 inhibitor EU-5346 induced cell death and decreased spheroid formation in Her2-positive BC cells. Of interest, EU-5346 induced ubiquitination of Mcl-1- bound Her2 demonstrating a previously unknown role for Mcl-1 to stabilize Her2 protein levels. Importantly, targeting Mcl-1 was also active in Her2-positive BC cells resistant to Her2 inhibitors, including a brain-primed Her2-positive cell line.

**Conclusion:**

Our data demonstrate a critical role of Mcl-1 in Her2-positive BC cell survival under hypoxic conditions and provide the preclinical framework for the therapeutic use of novel Mcl-1- targeting agents to improve patient outcome in BC.

**Electronic supplementary material:**

The online version of this article (doi:10.1186/s13058-016-0686-4) contains supplementary material, which is available to authorized users.

## Background

Breast cancer (BC) is the most common malignancy and the second most common cause of cancer-related mortality in women. The clinically relevant classification of BC is based on its histopathological appearance, tumor grade, lymph node involvement, and immunohistochemical properties including the presence of the estrogen receptor (ER), the progesterone receptor (PR), human epidermal growth factor receptor 2 (Her2; ErbB2, c-erbB2), and Ki-67 [1, 2]. Importantly, BC is known to be highly heterogenic with different intrinsic molecular subtypes. An additional BC classification based on molecular signatures has been developed recently with a clinical impact not only for patients’ prognosis but also the choice of treatment. Gene expression profiling has been utilized to classify BC into four different groups [3–6]. Specifically, BC is subgrouped into Luminal A, Luminal B, Her2-enriched, and basal-like BC [4]. Luminal-A BC, the most common subtype, is ER-positive and is characterized by the absence of Her2 expression, a low rate of proliferation (Ki67), and histologically low-grade tumors. Moreover, Luminal-A BC presents with high expression in mRNA and protein levels of the Luminal expression signature (e.g., *ESR1*, *GATA3*, *FOXA1*, *XBP1*, and *MYB*) [5, 7]. Luminal-B BC includes ER-positive but also ER-negative histologically high-grade tumors. In contrast to Luminal A, Luminal-B BC has an increased expression not only of proliferation genes such as *KI67* and cyclin B1 but also frequently overexpresses epidermal growth factor receptor (EGFR) and *HER2* [7]. Basal-like BC or triple-negative breast cancer (TNBC) is characterized by ER-negative, PR-negative, and Her2-negative tumors with high frequency of *TP53* (80 %) and *PIK3CA* (9 %) mutation [5]. Her2-positive (Her2-enriched) BC is characterized by the amplification of the *HER2* gene and other genes of the *HER2* amplicon including *STARD3* and *GRB7* [6, 8]. Her2 in particular acts as a coreceptor and enhances signaling pathways of other Her family members. It is activated by homodimerization with Her2 or heterodimerization with other Her family members. Importantly, the heterodimer between Her2 and Her3 has the highest mitogenic potential [9]. Overexpression of Her2 occurs in 15–20 % of BC patients and is associated with worse biologic behavior; that is, increased rate of metastasis, and poor clinical outcome without Her2-targeted treatment [10]. Unprecedented therapeutic advances have been achieved during the last years by combining Her2 inhibitors trastuzumab (Herceptin®, Genentech, South San Francisco, CA, USA), lapatinib (Tykerb®, GlaxoSmithKline, Philadelphia, PA, USA; Tyverb®, GlaxoSmithKline, London, UK), and pertuzumab (Perjeta®, Genentech, South San Francisco, CA, USA) with chemotherapeutic regimens, and by the introduction of ado-trastuzumab emtansine (T-DM1, Kadcyla®, Genentech, South San Francisco, CA, USA) monotherapy. However, inherent and acquired resistance to these agents remains a significant barrier to further reduce mortality in this BC patient subtype, highlighting the urgent need for novel therapies [11]. In addition, these drugs do not penetrate the blood–brain barrier as easily as they reach the rest of the body, with lapatinib in combination with capecitabine and T-DM1 monotherapy being a possible exception [12].

Hypoxic conditions develop during cancer progression because of rapidly proliferating tumor cells that reduce oxygen diffusion and impair perfusion of abnormal blood vessels in the tumor microenvironment. Cellular adaptation to hypoxia is predominantly mediated through protein stabilization of hypoxia-inducible factor (Hif) subunits. In BC, hypoxic regions have the potential to confer chemotherapy and radiation therapy resistance [13]. Molecular mechanisms which lead to the adaptation of BC cells to hypoxia are largely unknown.

Myeloid cell leukemia-1 (Mcl-1) is an anti-apoptotic protein of the Bcl-2 family characterized by its ability to oppose several apoptotic stimuli, a short half-life, its wide intracellular localization, and multiple pathways which tightly regulate Mcl-1 transcription, translation, and degradation [14]. Structurally, the N-terminus differs from the other anti-apoptotic Bcl-2 proteins in that it contains two polypeptide sequences enriched in proline, glutamic acid, serine, and threonine (PEST) [15]. PEST regions are made responsible for Mcl-1 degradation via the proteasome pathway, localization, and phosphorylation, thus providing the mechanistic base for the fine-tuned Mcl-1 protein functions in response to environmental stimuli and the cellular origin [14, 16, 17]. The pro-survival function of Mcl-1 is predominantly mediated by its binding to Bak and Bim. Conversely, release of Bak or Bim from their interaction with Mcl-1 induces apoptosis [18]. In addition, binding to the Bcl-2 homology 3 (BH3)-only protein NOXA enhances Mcl-1 degradation followed by caspase activation. In contrast, blockade of NOXA induction results in the suppression of apoptosis [19, 20]. Several past studies indicated that Mcl-1 is an important cancer target. Specifically, Mcl-1 is amplified in many cancers including BC and elevated Mcl-1 levels in BC correlate with increased tumor cell survival, growth, and poor prognosis [21–23]. Both epidermal growth factor and estrogen induce Mcl-1 expression via binding to their respective receptors and activation of downstream signaling pathways [24, 25]. Importantly, Mcl-1 mediates resistance against widely used anticancer therapies including paclitaxel [26] and gemcitabine [27] as well as early clinical BH3 mimetic drugs that block Bcl-2 and Bcl-xL [21, 28]. Moreover, trastuzumab sensitizes Her2-overexpressing cells to apoptosis by reducing anti-apoptotic Mcl-1 expression [29]. Based on these data, Mcl-1 holds great promise as a high-priority therapeutic target. Indeed, Mcl-1 is the current focus of widespread cancer drug development efforts, and a number of Mcl-1 inhibitors are in the cancer drug development pipeline worldwide [30].

In this study we determined a critical role of Mcl-1 in Her2-positive BC cell survival under hypoxic conditions also including brain-primed BC cells, and we present EU-5346 as a promising novel anti-Mcl-1 targeting agent. We thereby provide the preclinical framework for the therapeutic use of novel Mcl-1-targeting agents to improve patient outcome in BC.

## Methods

### Materials

Lapatinib ditosylate and ABT-199 were purchased from Santa Cruz Biotechnology (Heidelberg, Germany); cobalt (II) chloride hexahydrate from Sigma Aldrich (Schnelldorf, Germany); trastuzumab (Herceptin®) from Roche (San Francisco, CA, USA); and ZVAD pan-caspase inhibitor z-VAD-fmk from Bachem (Bubendorf, Switzerland). Antibodies against human Mcl-1 (S-19), extracellular signal-regulated kinase 2 (ERK2), and Ubiquitin (P4D1) were obtained from Santa Cruz Biotechnology; antibodies against Her2/ErbB2 (D8F12), Poly (ADP-ribose) polymerase (PARP), Bcl-xL, and Prohibitin-1 from Cell Signaling Technology (Boston, MA, USA); the antibody against α-tubulin from Sigma Aldrich; the antibody against Hif-1α, EpCAM, and ErbB2/Her2 from BD Biosciences (Heidelberg, Germany); the antibody against Bcl-2 (ab18210) from Abcam (Boston, MA, USA); and the antibody against murine Mcl-1 from Rockland Immunochemicals Inc., Limerick, PA 19468, USA.

### Isolation of primary tumor cells

BC cells were collected by centrifugation of pleural fluid. The cell pellet was resuspended with RPMI medium and placed in culture flasks; >95 % purity of BC cells was confirmed using anti-EpCAM and anti-Her2 antibodies by fluorescence-activated cell sorting (FACS) analysis. Samples were isolated from patients with advanced disease and resistant against multiple agents including hormone therapy, Her2 inhibitors, capecitabine, paclitaxel, eribulin, and everolimus. The collection and use of patient BC cells has been approved by the ethics committee of the Medical Faculty, University of Heidelberg (approval number 022/2013). Informed consent was obtained in accordance with the Declaration of Helsinki.

### Cell culture and conditions

MCF-7 cells were a kind gift from Dr Beckhove (German Cancer Research Center (DKFZ), Heidelberg, Germany), MDA MB-157 and MDA MB-468 cells were from Dr Oskarsson (DKFZ), HCC-1954 and MCF-10A cells were from Dr Wiemann (DKFZ), and SKBR3 cells were from Dr Trumpp (DKFZ). JIMT-1-BR3 cells (brain metastatic BC cell line) were a kind gift from Dr Steeg (Laboratory of Molecular Pharmacology, NCI’s Women's Malignancies Branch, National Cancer Institute/National Institutes of Health, Bethesda, MD, USA). JIMT-1-BR3 cells were obtained by a method detailed in previous publication [31]. All other cell lines were purchased from the Leibniz Institute DSMZ—German Collection of Microorganisms and Cell Cultures (Braunschweig, Germany). MCF-7, MDA MB 231, and HCC-1954 cells were cultured in RPMI 1640 medium (Gibco, Life Technologies, Grand Island, NY, USA) supplemented with 10 % heat-inactivated fetal bovine serum (FBS; PAA Laboratories, Cölbe, Germany), 1 % penicillin/streptomycin and 2 mM l-glutamine (all from Gibco, Life Technologies). MDA MB 453, MDA MB-157, MDA MB-468, JIMT-1, and JIMT-1 BR3 cells were cultured in Dulbecco’s modified Eagle’s medium (DMEM; Gibco, Life Technologies) supplemented with 10 % heat-inactivated FBS, 1 % penicillin/streptomycin and 2 mM l-glutamine. BT-474 and SKBR3 cells were maintained similarly to MDA MB 453 cells, but with the addition of minimum essential medium (MEM) nonessential amino acids (Gibco, Life Technologies). MCF-10A cells were maintained in DMEM/F12 supplemented with 10 % heat-inactivated FBS, 1 % penicillin/streptomycin, 2.5 mg insulin, 5 mg hydrocortisone (Sigma), 8 μl cholera toxin, and 10 μg human epidermal growth factor (Sigma Aldrich). Murine embryonic fibroblast (MEF) cell lines Mcl-1^wt/wt^ and Mcl-1^Δ/null^were generated by SV40 large T transformation followed by Tet-Cre-mediated deletion. Single cell clones were selected and then grown in DMEM supplemented with 10 % heat-inactivated FBS, 1 % penicillin/streptomycin, 2 mM l-glutamine, 2-mercaptoethanol (Sigma Aldrich), and MEM nonessential amino acids (Gibco, Life Technologies) from early passages. Both Mcl-1^wt/wt^ and Mcl-1^Δ/null^ MEFs were extensively characterized as being hypersensitive to various death stimuli with restorable resistance upon re-expression of Mcl-1 [32]. For hypoxia, cells were incubated in a hypoxia chamber (COY Laboratory Products, Ann Arbor, MI, USA) with a computerized oxygen controller to maintain an atmosphere of 0.5– 1 % oxygen, 5 % carbon dioxide, and 37 °C.

### Small interfering RNAs and cell transfection

BC cells were transiently transfected with indicated amounts of small interfering RNA (siRNA) siGENOME SMART pools of *MCL1* (4170) and *HER2* (2064), or the nontargeting control (mock) siRNA (Upstate Cell Signaling Solutions/Dharmacon RNA Technologies, Lafayette, CO, USA) using Lipofectamine® 2000 according to the manufacturer's instructions (Invitrogen, Darmstadt, Germany). Nontargeting control (mock) siRNA is composed of a pool of four siRNAs, which have been characterized by genome-wide microarray analysis and found to have minimal off-target signatures.

### Cell death assays

The inhibitory effect of hypoxia, si*MCL1*, or si*HER2* on BC cell lines was assessed using 3-(4,5-dimethylthiazol-2-yl)-2,5-diphenyl tetrazolium bromide (MTT; Sigma Aldrich) as described previously [33], or AlamarBlue^®^ assay (Invitrogen) according to the manufacturer's instructions.

### Cell lysis, immunoblotting, and immunoprecipitation

Treated or untreated cells were washed three times with phosphate-buffered saline and lysed with either lysis buffer (10 mm Tris, 50 mm NaCl, 1 % Triton, 30 mM sodium pyrophosphate, pH 7.05) or radioimmune precipitation assay lysis buffer (150 mM NaCl, 10 mM Tris, pH 7.2, 0.1 % SDS, 1 % Triton X-100, 1 % deoxycholate, 5 mM ethylenediamine tetraacetic acid) supplemented with Halt Protease and Phosphatase Inhibitor Cocktail (Pierce, Darmstadt, Germany). Insoluble material was removed by centrifugation (15,000 rpm for 30 minutes at 4 °C). Immunocomplexes were collected following overnight incubation at 4 °C with 10–20 μl of 100 % protein A-Sepharose® CL-4B beads (Amersham, Arlington Heights, USA). For western blotting, cell lysates (10–100 μg/lane) or immunoprecipitates (300–700 μg total proteins) were separated by 8 or 10 % SDS-PAGE prior to electrophoretic transfer onto Hybond™-C super nitrocellulose membranes (Amersham). After blocking with 5 % nonfat milk in phosphate-buffered saline-Tween®20 buffer at room temperature for 1 hour, membranes were sequentially blotted with the indicated specific primary antibodies and then with horseradish peroxidase-conjugated secondary mouse or rabbit Abs (Santa Cruz Biotechnology (Heidelberg, Germany)) and were developed using chemiluminescence (Amersham, Arlington Heights, USA) [33].

### Reverse transcription PCR

Reverse transcription PCR was performed as described previously [34]. Specifically, total RNA was purified with the RNeasy® Mini Kit (Qiagen, Hilden, Germany), according to the manufacturer's instructions, and then reverse transcribed and synthesized to cDNA using Omniscript® reverse transcriptase (Qiagen). PCR amplification was performed using Taq DNA polymerase (Qiagen). The primers used to amplify human *MCL1* were 5′-ATCTCTCGGTACCTTCGGGAGC-3′ (sense) and 5′-CCTGATGCCACCTTCTAGGTCC-3′ (antisense). The primers for human *β-ACTIN * were 5′-CTGGGACGACATGGAGAAAA-3′ (sense) and 5′-AAGGAAGGCTGGAAGAGTGC-3′ (antisense). The thermal cycle profile consisted of denaturing at 94 °C for 45 seconds, annealing at 60 °C for 45 seconds, and extension at 72 °C for 40 seconds. The samples were amplified for 30 cycles. We used 8 pmol of primers. The integrity of mRNA of all samples was confirmed by amplification of β-actin. PCR products were separated on a 1 % agarose gel and photographed.

### Mitochondrial isolation

Extracts of cytoplasmic and mitochondrial fractions were prepared using the Qproteome Mitochondria Isolation Kit (Qiagen), according to the manufacturer’s instructions.

### Proliferation assay

Proliferation was measured by the incorporation of 0.5 μCi/well [^3^H]-thymidine (Perkin Elmer, Baesweiler, Germany) during the last 8 hours of 72-hour experiments. Radioactive labeling was determined by harvesting the cells onto glass-fiber filtermates (Perkin Elmer) with an automatic cell harvester (Harvester 96, Tomtec Inc., Hamden, CT, USA) and counting using the Wallac Trilux Betaplate scintillation counter (Perkin Elmer).

### Annexin V apoptosis detection

Cells were treated as indicated, then washed with phosphate-buffered saline and costained with fluorescein isothiocyanate (FITC)-labeled Annexin V and propidium iodide (PI) using the FITC Annexin V Apoptosis Detection Kit I (BD Pharmingen, San Diego, CA, USA) following the manufacturer’s instructions. Apoptosis was analyzed on a FACS Canto II flow cytometer (BD Biosciences).

### Spheroid formation assay

Single multicellular BC spheroids were formed as described previously [35]. Briefly, 4 × 10^3^–6 × 10^3^ BC cells were seeded on agarose-coated (Sigma Aldrich, Steinheim, Germany) 96-well plates. At the end of the experiments, spheroids were stained with Calcein AM (green) (Sigma Aldrich, Steinheim) and PI (red) (Sigma Aldrich, Steinheim). For image capturing, the Olympus CKX41 microscope using 4× and 10× magnification was connected to an Olympus XC30 digital camera and exported to the cellSens standard software (version 1.4) (Olympus Corporation, Shinjuku, Tokyo, Japan). Based on the automatic measurement of the radius, volumes of the spheroids were calculated (V = 4/3 πr^3^).

### Isobologram analysis

The interaction between EU-5346 and trastuzumab was analyzed using the CompuSyn software program (ComboSyn, Inc., http://www.combosyn.com/), which is based on the Chou-Talalay method [36]. When combination index (*CI*) = 1, this equation represents the conservation isobologram and indicates an additive effect. *CI* <1 indicates synergism; *CI* >1 indicates antagonism.

### Statistical analysis

Statistical significance of differences observed in treated versus control cultures was determined by means of an unpaired Student *t* test. Statistical analyses were performed using SigmaPlot version 12.5 software (Systat Software GmbH, D-40699 Erkrath, Germany). The minimal level of significance was *p* <0.05*.*

## Results

### Mcl-1 expression correlates with improved adaptation of Her2-positive BC cell lines to hypoxia

**Fig. 1:**
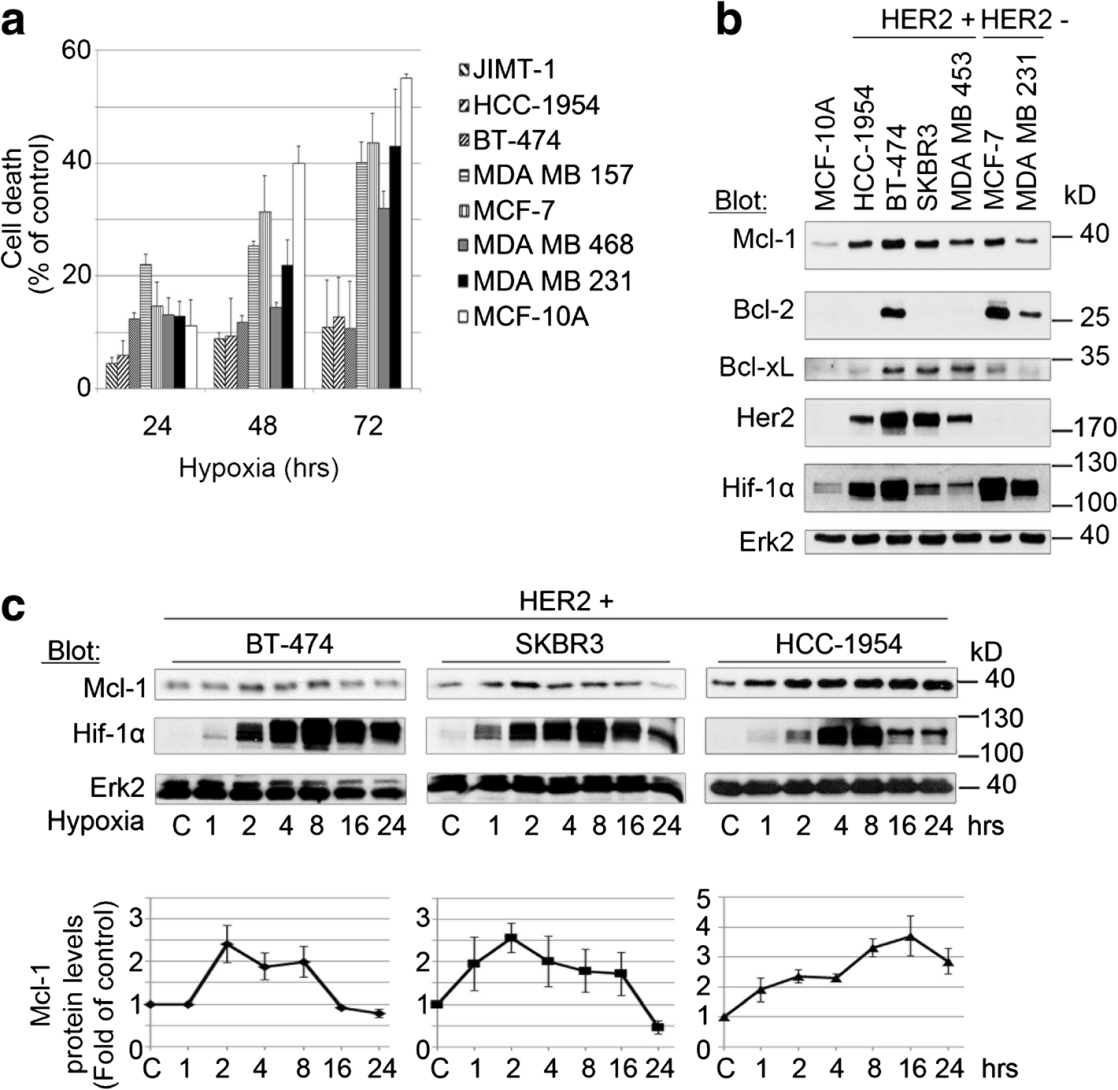
Mcl-1 expression correlates with improved adaptation of Her2-positive BC cell lines to hypoxia. **a** Improved adaptation to hypoxic conditions of Her2-positive versus Her2-negative BC cells and the benign MCF-10A cell line. BC cell lines were incubated under hypoxic conditions for the time periods indicated. Cell survival was analyzed by MTT assay. Data represent mean ± SD for triplicate samples. Results shown are representative of three independent experiments. **b** Significant protein levels of Mcl-1, but not Bcl-2 or Bcl-xL, in BC cells under hypoxic conditions. BC cell lines or the nonmalignant MCF-10A cells were incubated for 4 hours under hypoxic conditions and whole-cell extracts were analyzed by immunoblotting with indicated antibodies. Immunoblotting for Erk2 confirmed equal protein loading. **c** Upregulation of Mcl-1 in Her2-positive BC cells under hypoxic conditions. BC cell lines were incubated under hypoxic conditions for up to 24 hours. Whole-cell extracts were analyzed by immunoblotting with indicated antibodies. Immunoblotting for Erk2 confirmed equal protein loading. Densitometric measurements (NIH ImageJ software) [59] were used to quantitate Mcl-1 protein levels from western blot images normalized for Erk2. Densitometric data represent mean values ± SD. Data shown are representative of three independent experiments. *C* normoxic control, *Erk2* extracellular signal-regulated kinase 2, *Her2* human epidermal growth factor receptor 2, *Hif* hypoxia-inducible factor, *Mcl-1* myeloid cell leukemia-1

Given the prognostic adverse role of hypoxia in BC, we first evaluated whether there are differences in the adaptation between cell lines representing different BC subtypes [37, 38] to hypoxia. Our results demonstrate improved adaptation of Her2-positive BC cells (BT-474, HCC-1954, JIMT-1) versus TNBC (MDA MB 231, MDA MB-468, MDA MB-157), Her2-negative cells (MCF-7), and a nonmalignant breast cell line (MCF-10A) to hypoxic conditions utilizing both MTT (Fig. 1a) and [^3^H]-thymidine uptake assays (data not shown). Previous studies have indicated a key role of the Bcl-2 family of anti-apoptotic proteins, and Mcl-1 in particular, in BC cell survival. Indeed, Her2 and Mcl-1 expression correlates in BC cells under normoxic conditions [29]. Whether Mcl-1 contributes to the adaptation of BC cells to hypoxia in general, and Her2-positive BC cells in particular, is currently unknown. Our results show consistently high Mcl-1, but variable Bcl-2 and Bcl-xL protein levels in a cell line panel of different BC subtypes, including Luminal A-like (MCF-7), Luminal B-like/Her2-positive (BT-474), Her2-positive (HCC-1954, SKBR3), and TNBC cells (MDA MB-231) [37, 38] under hypoxic conditions (Fig. 1b). In contrast, Mcl-1 protein levels were low in the benign MCF-10A BC cell line. Moreover, Mcl-1 protein levels of BC exposed to hypoxia were transiently increased 2.41-fold (standard deviation (SD) ±0.44), 2.57-fold (SD ±0.34), and 3.31-fold (SD ±0.31) in Her2-positive BT474, SKBR3, and HCC-1954 BC cells, respectively. Increases in Mcl-1 protein levels correlated with increases in protein levels of Hif-1α (Fig. 1c). To determine whether changes in Mcl-1 protein levels were due to elevated transcription or post-transcriptional regulation, we next detected *MCL1* mRNA levels over a period of 24 hours using semiquantitative reverse transcription PCR. Our results show that mRNA levels of *MCL1* did not change over time, indicating a transient hypoxia-mediated increase in Mcl-1 stability (Additional file 1: Figure S1). Taken together, these data suggest a role for Mcl-1 in BC cell adaptation to hypoxia in general, and Her2-positive BC cells in particular.

### Mcl-1 is an upstream regulator of Her2 under hypoxic conditions

**Fig. 2:**
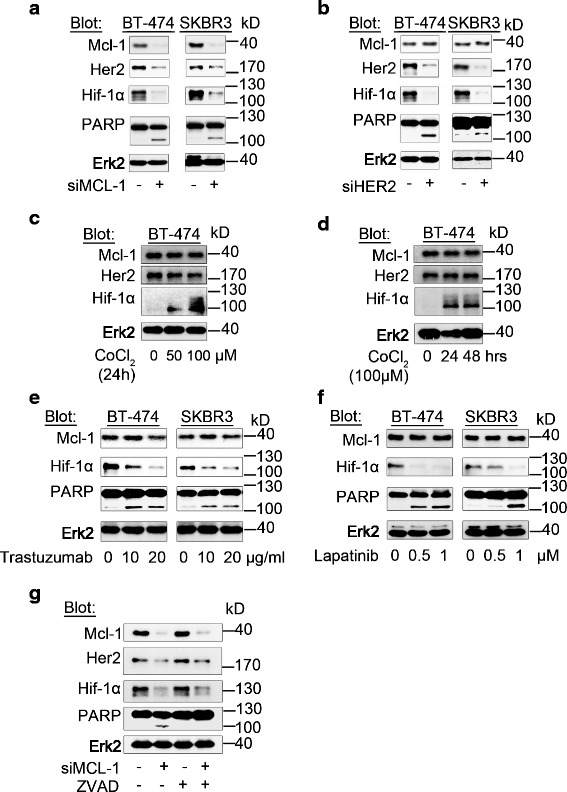
Mcl-1 is an upstream regulator of Her2 under hypoxic conditions. **a** Genetic depletion of Mcl-1 in Her2-positive BC cells promotes downregulation of Her2 and Hif-1α followed by inhibition of BC cell survival. **b** Mcl-1 protein levels do not decrease after genetically downregulating Her2. **a**, **b** BC cells were transfected with *siMCL1* **a** or *siHER2* **b** for 2 days and then exposed to hypoxia for 6 hours. **c**, **d** CoCl_2_-mediated stabilization of Hif-1α does not alter Mcl-1 or Her2 levels. BT-474 cells were exposed to CoCl_2_with indicated doses for 24 hours **c** and 100 μM CoCl_2_ for the indicated time periods **d**. **e**, **f** Pharmacologically targeting Her2 decreases Hif-1α but not Mcl-1 protein levels. BC cells were treated with the indicated doses of trastuzumab **e** or lapatinib **f** for 2 days and then exposed to hypoxia for 6 hours. **g** *siMCL1*-induced downregulation of Her2 and Hif-1α is not triggered by caspase-mediated off-target effects of *MCL1* siRNA. Her2-positive BC cells were transfected with *siMCL1* for 2 days and then exposed to hypoxia for 6 hours. ZVAD (50 μM) was added 24 hours before collecting samples. **a**–**g** Whole-cell extracts were analyzed by immunoblotting with indicated antibodies. Immunoblotting for Erk2 confirmed equal protein loading. *Erk2* extracellular signal-regulated kinase 2, *Her2* human epidermal growth factor receptor 2, *Hif* hypoxia-inducible factor, *kD* kilodalton, *Mcl-1* myeloid cell leukemia-1, *PARP* Poly (ADP-ribose) polymerase

To evaluate a potential functional interrelation of Mcl-1 and Her2 in Her2-positive BC cells under hypoxic conditions, we next transfected the Her2-positive BC cell lines BT-474 and SKBR3 with a pool of siRNA directed against the *MCL1* gene (*siMCL1*) and tested for both Her2 and Hif-1α protein levels. Our data demonstrate marked downregulation of Her2 and Hif-1α in Her2-positive BC cells depleted of Mcl-1. Moreover, silencing of *MCL1* induced cell death as evidenced by PARP cleavage (Fig. 2a). Surprisingly, in contrast to previous reports which show Her2-dependent Mcl-1 regulation under normoxic conditions [24, 25, 29], siRNA-mediated knockdown of the *HER2* gene (*siHER2*) in Her2-positive BC cells did not affect Mcl-1 protein levels under hypoxic conditions (Fig. 2b). CoCl_2_ binds to the iron center of the Hif-specific hydroxylase. It thereby inhibits the hydroxylation of Hif, its ubiquitination by pVHL, and proteasomal degradation. In agreement with our hypothesis that Mcl-1 regulates Her2 and downstream Hif-1α protein levels in BC cells under hypoxic conditions, treatment with CoCl_2_ increased Hif-1α protein levels in a dose-dependent (Fig. 2c) and time-dependent (Fig. 2d) manner, without changing Mcl-1 or Her2 levels. Moreover, treatment of Her2-positive BC cells but not Her2-negative BC cells with the Her2 inhibitors trastuzumab (Fig. 2e) and lapatinib (Fig. 2f) under hypoxic conditions induced marked downregulation of Hif-1α, but not Mcl-1. These data correlated with the induction of cell death, as evidenced by PARP cleavage (Fig. 2e,f). Importantly, treatment with the pan-caspase inhibitor Z-VAD-FMK abrogated PARP cleavage induced by *siMCL1*, but not the downregulation of Her2 and Hif-1α in BC cells. These data exclude a caspase-mediated off-target effect of *siMCL1* on Her2 protein levels (Fig. 2g). Taken together, these data confirm a role for Mcl-1 upstream of Her2 and Hif-1α under hypoxic conditions.

### The novel small molecule inhibitor EU-5346 specifically blocks Mcl-1 followed by cell death

**Fig. 3:**
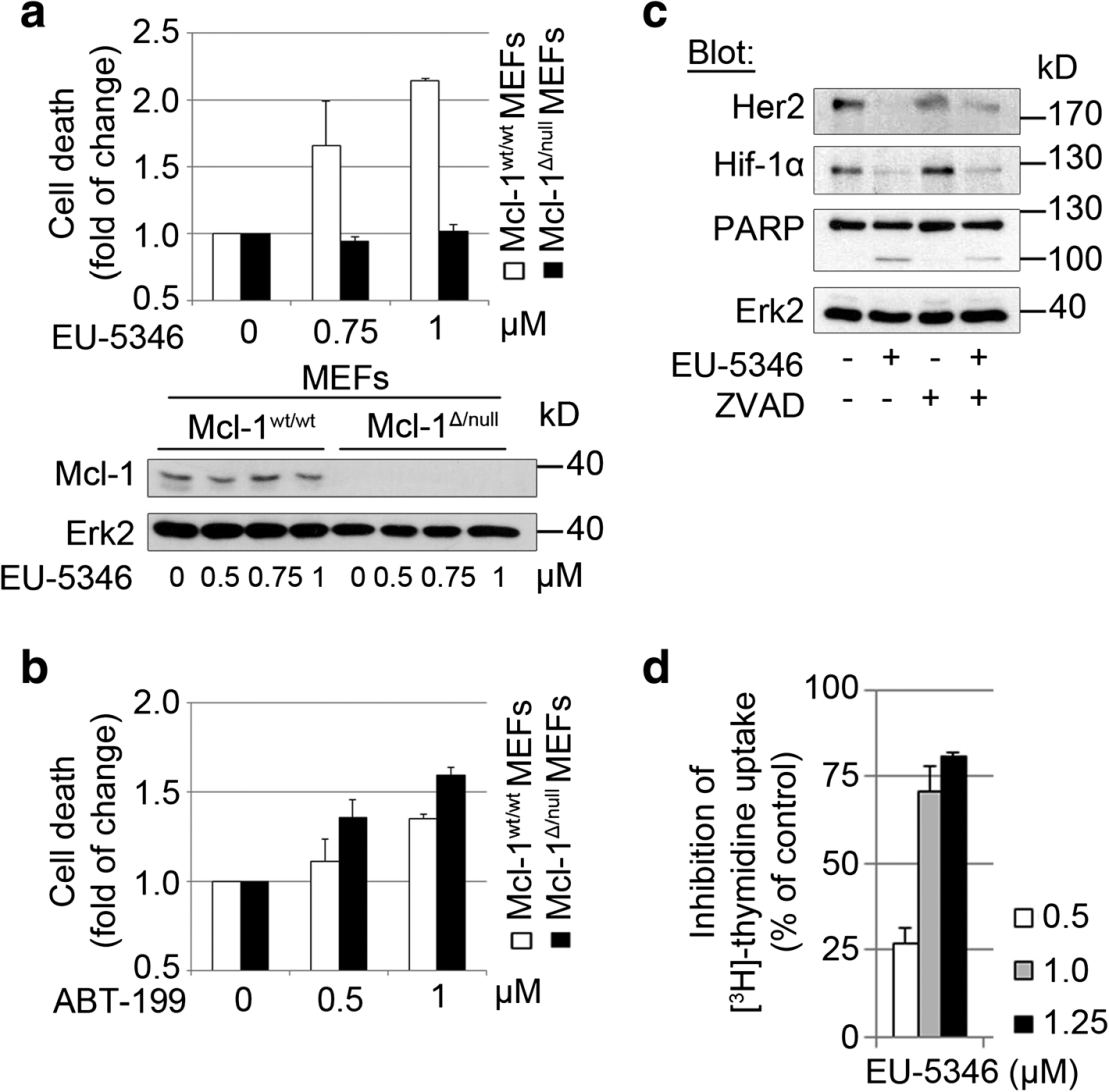
The novel small molecule inhibitor EU-5346 specifically blocks Mcl-1 followed by cell death. **a**, **b** EU-5346 induces apoptosis in Mcl-1^wt/wt^ but not Mcl-1^Δ/null^ MEFs **a**. In contrast, ABT-199 induces apoptosis in both Mcl-1^wt/wt^ and Mcl-1^Δ/null^ MEFs **b**. MEFs were treated with indicated concentrations of EU-5346 **a** or ABT-199 **b** for 72 hours prior to Annexin V and PI staining. Data represent mean ± SD for triplicate samples. Results shown are representative of three independent experiments. **c** EU-5346-induced downregulation of Her2 and associated cell death are not triggered by caspase-mediated off-target effects of Mcl-1 siRNA. Her2-positive BC (SKBR3) cells were treated with EU-5346 and/or ZVAD (50 μM) and exposed to hypoxia during the last 6 hours. **d** EU-5346 inhibits proliferation of Her2-positive BC cells in a dose-dependent manner. BC cells were treated with EU-5346 for 3 days under hypoxic conditions. [^3^H]-thymidine was added during the last 8 hours. Data represent mean ± SD for triplicate samples. Results shown are representative of three independent experiments. **c**, **d** Whole-cell extracts were analyzed by immunoblotting with indicated antibodies. Immunoblotting for Erk2 confirmed equal protein loading. *Erk2* extracellular signal-regulated kinase 2, *Her2* human epidermal growth factor receptor 2, *Hif* hypoxia-inducible factor, *kD* kilodalton, *Mcl-1* myeloid cell leukemia-1, *MEF* murine embryonic fibroblast, *PARP* Poly (ADP-ribose) polymerase

Our own and other studies show that blocking the effects of Mcl-1 is a promising approach to slow tumor growth, induce apoptosis, and overcome drug resistance in BC cells, and Her2-positive BC cells in particular. One approach to target Mcl-1 functions is the inhibition of its interaction with pro-apoptotic oncogenes of the Bcl-2 family. Using ultrahigh-throughput screening of 315,000 compounds coupled with the hit optimization strategy, the hydroxyquinoline-derived, small molecule EU-5346 (also ML311; Eutropics Pharmaceuticals, Cambridge, MA, USA) was identified as a potent inhibitor of Mcl-1/Bim interaction that shows selective activity against Mcl-1 primed cells [39]. In contrast, only the Bcl-2-specific BH3-mimetic navitoclax but not EU-5346 caused cell death in highly primed Bcl-2 cells [40]. Our own results demonstrate that EU-5346 induces apoptosis in Mcl-1^wt/wt^ but not in Mcl-1^Δ/null^ MEFs (Fig. 3a). Moreover, EU-5346 induced cell death in Bcl-2/Bcl-xL double knockout cells that were established as having Mcl-1 as the dependent anti-apoptotic Bcl-2 family protein [40]. Having verified the presence of Bcl-2 (Additional file 1: Figure S2a), the potent and highly selective Bcl-2 inhibitor ABT-199 [41] induced apoptosis in both Mcl-1^wt/wt^ and Mcl-1^Δ/null^ MEFs (Fig. 3b). These data indicate the specificity of EU-5346 against Mcl-1. Similar to siRNA-mediated knockdown of Mcl-1, EU-5346 induced marked downregulation of Her2 and Hif-1α in SKBR3 BC cells followed by cell death, as evidenced by PARP cleavage (Fig. 3c) and inhibition of [^3^H]-thymidine uptake (Fig. 3d). Treatment with the pan-caspase inhibitor Z-VAD-FMK abrogated PARP cleavage induced by EU-5346-mediated inhibition of Mcl-1, but not the downregulation of Her2 and Hif-1α in BC cells (Fig. 3c). Moreover, semiquantitative reverse transcription PCR did not show EU-5346-induced changes of mRNA levels of Mcl-1, supporting its role as a BH3-mimetic (Additional file 1: Figure S2b). Taken together these data strongly indicate a role for EU-5346 as a specific inhibitor of Mcl-1 in Her2-positive BC cells.

### Her2 co-localizes with Mcl-1 within the mitochondrial fraction

**Fig. 4:**
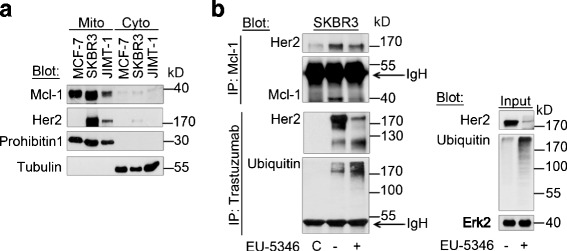
Her2 colocalizes with Mcl-1 within the mitochondrial fraction. **a** Mcl-1 and Her2 colocalize within the mitochondrial fraction. Mitochondrial and cytosolic proteins were isolated using the Qproteome Mitochondria Isolation Kit (Qiagen) according to the manufacturer’s instructions and analyzed by immunoblotting with indicated antibodies. Anti-Prohibitin 1 was used as a mitochondrial marker and anti-tubulin was used as a cytoplasmic marker. BC cells were exposed to hypoxia for 6 hours. **b** EU-5346 induces ubiquitination of Mcl-1-bound Her2. SKBR3 cells were treated with EU-5346 for 3 days and exposed to hypoxia during the last 6 hours. Whole-cell extracts were immunoprecipitated (*IP*) with either Mcl-1 antibody or trastuzumab and analyzed by immunoblotting with indicated antibodies. Nonspecific protein binding and detection were excluded by incubating protein A-Sepharose beads with lysis buffer and Mcl-1 antibody only (*left panel*). Input (*right panel*). *C* control, *Erk2* extracellular signal-regulated kinase 2, *Her2* human epidermal growth factor receptor 2, *IgH* heavy chain, *kD* kilodalton, *Mcl-1* myeloid cell leukemia-1

To further support the proposed functional interrelation of Mcl-1 and Her2, we next explored whether Mcl-1 interacts with Her2 under hypoxic conditions. It is well established that Her2 localizes to the cell membrane where it phosphorylates downstream substrates on their tyrosine residues in response to extracellular stimulation. Surprisingly, recent studies found that Her2 also localizes to the mitochondrial fraction of BC cells, where it promotes resistance to hypoxia and E-twenty-six (ETS) transcription factor inhibitors [42]. We therefore next sought to investigate whether Her2 and Mcl-1 colocalize in the mitochondrial fraction. Indeed, using western blot analysis our results show colocalization of Mcl-1 and Her2 in the mitochondrial fraction. Probing of western blot membranes with antibodies against prohibitin-1 and α-tubulin secured the high purity of the mitochondrial fraction (Fig. 4a).

Importantly, our results show that Mcl-1 coimmunoprecipitates with Her2 (Fig. 4b) and that Mcl-1 increases the stability of Her2 by modulating the Her2 protein degradation mechanism. Increased ubiquitination of Her2 was more pronounced in *siMCL1*-treated (data not shown) and EU-5346-treated BC cells than in control cells (Fig. 4b). Receptor internalization is often associated with receptor ubiquitination and targeting to proteasomes for degradation [43]. Indeed, besides increased Her2 ubiquitination, treatment of SKBR3 cells with EU-5346 (Fig. 4b) as well as *siMCL1* (data not shown) resulted in the formation of a 125–130 kD fragment of Her2 that was associated with a reduction in full-length Her2.

Taking these results together, the specific Mcl-1 inhibitor EU-5346 decreases Her2 protein levels. Functionally, our data show that the interaction of Mcl-1 and Her2 mediates protection of Her2 ubiquitination and subsequent degradation. Conversely, blockade of Mcl-1 activity increases Her2 ubiquitination and degradation.

### Synergistic effects of combining trastuzumab with Mcl-1-targeting approaches in trastuzumab-sensitive Her2-positive BC cells

**Fig. 5:**
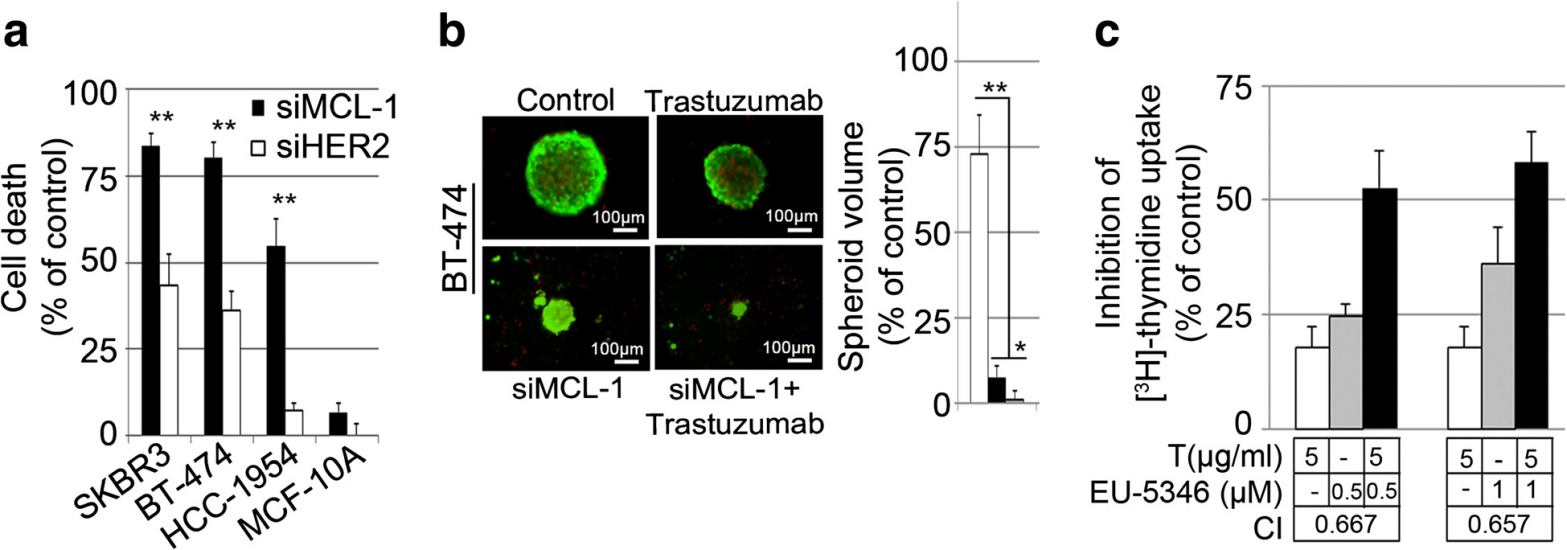
Synergistic effects of combining trastuzumab with Mcl-1-targeting approaches in trastuzumab-sensitive Her2-positive BC cells. **a** Genetic depletion of Mcl-1 or Her2 induces cell death in Her2-positive BC cells, but not benign breast cells (MCF-10A). BC and MCF-10A cells were transfected with *siMCL1* (*filled bars*) or siHER2 (*open bars*) for 30 hours and then exposed to hypoxia for 2 days. Cell survival was determined by AlamarBlue^®^ assay. Data represent mean ± SD for triplicate samples. Results shown are representative of three independent experiments. **b** Synergistic effects of combining trastuzumab with *siMCL1* in Her2-positive BC cells. BT-474 cells (5.5 × 10^3^ cells) were seeded per well in agar-coated 96-well plates prior to *siMCL1* transfection. Spheroid formation was assessed in *siMCL1*-treated and/or trastuzumab-treated and control cells using an inverted fluorescence light microscope. Photographs (10× magnification) of spheroid formation are representative of each group and three independent experiments (*left*). Spheroid volumes were calculated as described in Materials and methods. Data represent mean ± SD (*right*). **c** Synergistic growth inhibition of trastuzumab and EU-5346 in trastuzumab-sensitive Her2-positive BC cells under hypoxic conditions. BT-474 cells were pretreated with trastuzumab or dimethyl sulfoxide (DMSO) for 24 hours under normoxic conditions followed by EU-5346 treatment or DMSO for 72 hours under hypoxic conditions. [^3^H]-thymidine was added during the last 8 hours. Data represent mean ± SD for triplicate samples. Results shown are representative of three independent experiments. Trastuzumab (*white bars*), EU-5346 (*gray bars*), and drug combination (*black bars*). Combination index (*CI*) was calculated using the Combosyn software as described in Materials and methods. **a**, **b** *p <0.05 and **p <0.001 by Student’s *t* test

In Her2 inhibitor-sensitive Her2-positive BC cells, *siMCL1* induced significantly higher rates of cell death when compared with *siHER2*, indicating the existence of Mcl-1-dependent survival pathways in Her2-positive BC cells, which are independent of the Mcl-1–Her2 axis (Fig. 5a). In agreement with these data, *siMCL1* significantly reduced spheroid formation in both BT-474 (Fig. 5b) and SKBR3 (data not shown) cells. Moreover, the combination therapy of trastuzumab with EU-5346 synergistically triggered cell death in BT-474 (Fig. 5c), as determined by [^3^H]-thymidine uptake and subsequent analysis according to the Chou-Talalay method.

Taken together, our results indicate the existence of Mcl-1-dependent survival pathways in Her2-positive BC cells, which are independent of the Mcl-1–Her2 axis; and support the therapeutic benefit of combining Her2 and Mcl-1 inhibitors.

### Genetically and pharmacologically targeting Mcl-1 induces cell death also in trastuzumab-resistant Her2-positive BC cells

**Fig. 6:**
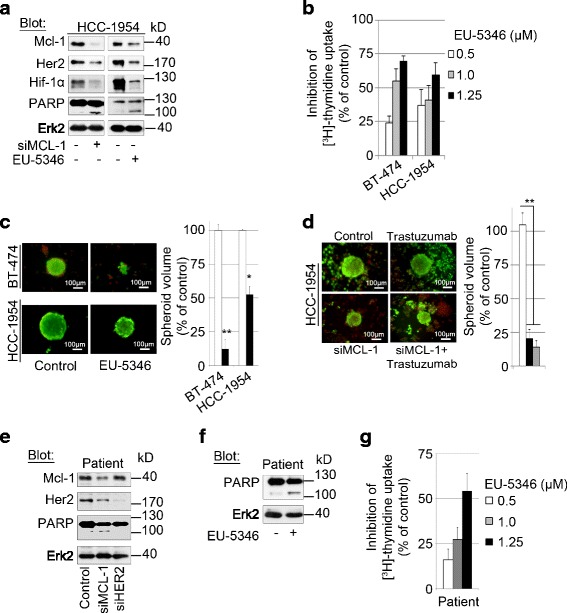
Genetically and pharmacologically targeting Mcl-1 induces cell death also in trastuzumab-resistant Her2-positive BC cells. **a** Both genetic depletion of Mcl-1 (*siMCL1*) and EU-5346 induces apoptosis in trastuzumab-resistant HCC-1954 BC cells. BC cells were transfected with *siMCL1* (*left*) for 2 days or treated with EU-5346 (*right*) for 3 days and then exposed to hypoxia for 6 hours. **b** EU-5346 inhibits proliferation in Her2 inhibitor-sensitive and inhibitor-resistant BC cells in a dose-dependent manner. **c** EU-5346 inhibits spheroid formation in both trastuzumab-sensitive (BT-474) and trastuzumab-resistant (HCC-1954) BC cells. **d** Combining trastuzumab with *siMCL1* in Her2 inhibitor-resistant Her2-positive BC cells does not induce synergistic effects. HCC-1954 cells (5.5 × 10^3^ cells) were seeded per well in agar-coated 96-well plates prior to *siMCL1* transfection. **c**, **d** Spheroid formation was assessed in EU-5346-treated or *siMCL1*-treated and/or trastuzumab-treated and control cells using an inverted fluorescence light microscope. Photographs of spheroid formation (10× magnification) are representative of each group and three independent experiments (*left*). Spheroid volumes were calculated as described in Materials and methods. Data represent mean ± SD (*right*). *p <0.05 and **p < 0.001 by Student’s *t* test. **e**, **f** *siMCL1* **e** and EU-5346 **f** but not *siHER2* **e** induces apoptosis in multidrug/Her2 inhibitor-resistant patient BC cells. BC cells were transfected with *siMCL1* or *siHER2* **e** for 2 days or treated with EU-5346 **f** for 3 days and then exposed to hypoxia for 6 hours. **a**, **e**, **f** Whole-cell extracts were analyzed by immunoblotting with indicated antibodies. Immunoblotting for Erk2 confirmed equal protein loading. **g** EU-5346 inhibits proliferation in multidrug/Her2 inhibitor-resistant patient BC cells in a dose-dependent manner. **b**, **g** BC cells were treated with EU-5346 for 3 days under hypoxic conditions. [^3^H]-thymidine was added during the last 8 hours. Data represent mean ± SD for triplicate samples. Results shown are representative of three independent experiments. *Erk2* extracellular signal-regulated kinase 2, *Her2* human epidermal growth factor receptor 2, *Hif* hypoxia-inducible factor, *kD* kilodalton, *Mcl-1* myeloid cell leukemia-1, *PARP* Poly (ADP-ribose) polymerase

All Her2-positive BC patients eventually develop resistance to trastuzumab and other Her2 inhibitors. The precise mechanism of resistance against these inhibitors remains elusive. We next investigated whether genetically and pharmacologically targeting Mcl-1 is also active in trastuzumab-resistant HCC-1954 cells. Similar to trastuzumab-sensitive BT-474 and SKBR3 BC cells (Figs. 2 and 3), both *siMCL1* and EU-5346 treatment induced cell death in HCC-1954 cells, as evidenced by the induction of PARP cleavage (Fig. 6a), inhibition of [^3^H]-thymidine uptake (Fig. 6b), and inhibition of spheroid formation (Fig. 6c, d). As expected, in contrast to BT-474 cells no synergistic effects of combining trastuzumab with *siMCL1* (Fig. 6d) or EU-5346 (data not shown) were observed on spheroid formation (Fig. 6d) or [^3^H]-thymidine uptake followed by the Cou-Talalay analysis (data not shown). Similar results were obtained in primary BC cells isolated from a patient with advanced disease and resistant against multiple agents including Her2 inhibitors, capecitabine, paclitaxel, eribulin, and everolimus. Specifically, *siMCL1* (Fig. 6e) and EU-5346 (Fig. 6f,g) but not *siHER2* (Fig. 6e) induced cell death as evidenced by PARP cleavage (Fig. 6e,f) and dose-dependent inhibition of [^3^H]-thymidine uptake (Fig. 6g).

Taken together these data demonstrate that targeting Mcl-1 has therapeutic potential not only in Her2 inhibitor-sensitive but also in Her2 inhibitor-resistant BC cells.

### Genetically and pharmacologically targeting Mcl-1 induces cell death in brain-primed Her2 inhibitor-resistant Her2-positive BC cells

**Fig. 7:**
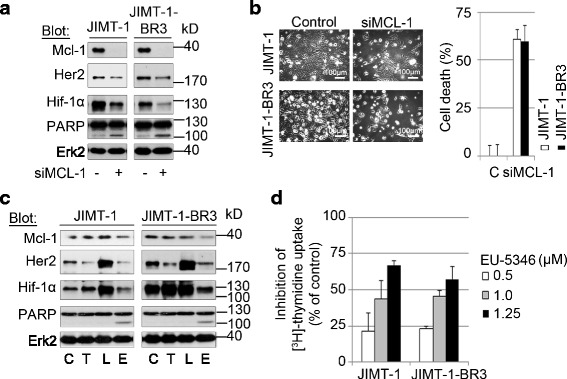
Genetically and pharmacologically targeting Mcl-1 induces cell death in brain-primed Her2 inhibitor-resistant Her2-positive BC cells. **a**, **b** Genetic depletion of Mcl-1 induces apoptosis in both maternal Her2 inhibitor-resistant Her2-positive JIMT-1 cells as well as in brain-primed JIMT-1 BR3 cells. **a** BC cells were transfected with *siMCL1* for 2 days and exposed to hypoxia for 6 hours. Whole-cell extracts were analyzed by immunoblotting with indicated antibodies. Immunoblotting for Erk2 confirmed equal protein loading. **b** BC cells were transfected with *siMCL1* for 30 hours and then exposed to hypoxia for 2 days. Cell survival was determined by AlamarBlue^®^ assay. Data represent mean ± SD for triplicate samples. Results shown are representative of three independent experiments. **c** EU-5346 overcomes Her2 inhibitor resistance in brain-primed Her2-positive BC cells. BC cells were treated with indicated drugs for 3 days and then exposed to hypoxia during the last 6 hours. Whole-cell extracts were analyzed by immunoblotting with indicated antibodies. Immunoblotting for Erk2 confirmed equal protein loading. **d** EU-5346 inhibits proliferation of brain-primed Her2-positive BC cells in a dose-dependent manner. BC cells were treated with EU-5346 for 3 days under hypoxic conditions. [^3^H]-thymidine was added during the last 8 hours. Data represent mean ± SD for triplicate samples. Results shown are representative of three independent experiments. *C* control (dimethyl sulfoxide), *E* EU-5346, *Erk2* extracellular signal-regulated kinase 2, *Her2* human epidermal growth factor receptor 2, *Hif* hypoxia-inducible factor, *kD* kilodalton, *L* lapatinib, *Mcl-1* myeloid cell leukemia-1, *T* trastuzumab

Brain metastasis is an end stage in BC progression. Compared with hormone receptor (HR)-positive BC patients, Her2-positive BC patients show a high incidence of brain metastasis of more than 20 % [44], with an overall survival of 4-6 months with whole brain radiotherapy to about 18 months with multimodal therapies [45]. Although trastuzumab is effective for systemic BC, its efficacy in brain metastasis remains controversial [46]. Moreover, the incidence of brain metastatic disease after adjuvant trastuzumab is increasing with the improved management of systemic disease and prolongation of survival [11, 47]. We next sought to investigate the anti-BC activity of targeting Mcl-1 in brain-primed (JIMT-1BR3) versus maternal Her2 JIMT-1 cells. Our data demonstrate marked downregulation of Her2 and Hif-1α in both JIMT-1 and JIMT-1BR3 cells after siRNA-mediated Mcl-1 downregulation (Fig. 7a). Moreover, silencing of Mcl-1 induced cell death as evidenced by PARP cleavage (Fig. 7a), and AlamarBlue^®^ assay (Fig. 7b). Importantly, similar to maternal JIMT-1 cells, EU-5346 but not trastuzumab and lapatinib induced cell death in brain-primed JIMT-1 BR3 cells, as evidenced by PARP cleavage (Fig. 7c) and [^3^H]-thymidine uptake (Fig. 7d). Taken together, these data indicate a therapeutic role of targeting Mcl-1 in BC brain metastasis.

## Discussion

The present study demonstrates a critical role of Mcl-1 predominantly in Her2-positive BC cell survival and proliferation under hypoxic conditions. Under normoxic conditions, Her2 regulates Mcl-1 upregulation. Conversely, trastuzumab treatment reduces Mcl-1 levels and renders cells sensitive to chemotherapy [29]. Surprisingly, in contrast to normoxia our data demonstrate a role for Mcl-1 upstream of Her2 in BC cells under hypoxic conditions. Functionally, we show that under hypoxic conditions Her2 is detected also in the mitochondrial fraction, where it binds Mcl-1, strongly indicating a functional interrelation of these two proteins in BC pathogenesis.

**Fig. 8:**
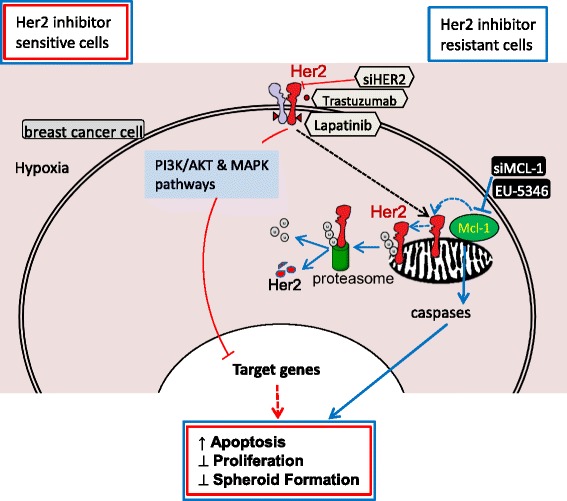
Summary: Mcl-1 confers protection of Her2-positive BC cells to hypoxia—therapeutic implications. Homodimerization of Her2 with Her2 or heterodimerization of Her2 with Her3 enhances phospho-inositol 3-kinase (*PI3K*)/AKT and RAS/mitogen-activated protein kinase (*MAPK*) pathways, which regulate BC cell proliferation, survival, and migration, as well as angiogenesis [60, 61]. The Her2 inhibitor trastuzumab [62] binds to the C-terminal portion of Her2. Another Her2 inhibitor, lapatinib [63], binds to the ATP binding site of Her2, but also Her1. Similar to *siHER2*, trastuzumab and lapatinib inhibit downstream signaling events, induce apoptosis, and inhibit proliferation of BC cells (*red lines*). Here we show a novel role for the antiapoptotic Bcl-2 family member Mcl-1 in Her2-positive BC cell adaptation to hypoxia. Specifically, our results show that Mcl-1 forms a protein complex with Her2 at the mitochondrial membrane and stabilizes Her2 by inhibiting its ubiquitination. Conversely, genetically (*siMCL1*) or pharmacologically (EU-5346) targeting Mcl-1 triggers ubiquitination and proteosomal degradation of Her2, thereby inducing apoptosis, and inhibiting proliferation and spheroid formation under hypoxic conditions. In addition, our results indicate the existence of a Mcl-1-dependent survival pathway in Her2-positive BC cells, which is independent of the Mcl-1–Her2 axis supporting the therapeutic benefit of combining Her2 (*red lines*) and Mcl-1 inhibitor (*blue lines*). Importantly, based on these findings, targeting Mcl-1 is also active in Her2-positive BC cells resistant to Her2 inhibitors, including a brain-primed Her2-positive cell line. *Her* human epidermal growth factor receptor, *Mcl-1* myeloid cell leukemia-1. (Color figure online)

To date, therapeutic BH3 mimetics that abrogate anti-apoptotic activity have been predominantly developed to target Bcl-2 and Bcl-xL. They function by slotting into the hydrophobic groove on the surface of Bcl-2 and Bcl-xL, thereby blocking their capacity to inhibit apoptosis [48]. Based on their promising preclinical activities, Bcl-2/Bcl-xL inhibitor ABT-737 [48] and its oral derivate navitoclax/ABT-263 [49], as well as the highly selective Bcl-2 inhibitor ABT-199 [41], are currently validated in advanced clinical trials in solid tumors, acute myeloid leukemia (AML), chronic lymphocytic leukemia (CLL), and non-Hodgkin lymphoma (NHL) (https://clinicaltrials.gov). Similar to previous data, our own results show consistently high Mcl-1 but variable Bcl-2 and Bcl-xL protein levels in cell lines of different BC subtypes. It is therefore clear that inhibitors of Mcl-1 hold great promise as a new class of targeting agents and are the current focus of widespread cancer drug development efforts. However, the clinical development of Mcl-1 inhibitors has been challenged by structural differences between the BH3-binding grooves of Mcl-1 and Bcl-2. Indeed, because of low affinities, ABT-737, navitoclax, and ABT-199 do not block Mcl-1 activity [28, 48, 49]. The high interest towards the identification of Mcl-1 inhibitors has resulted in the recent development of several small molecule BH3 mimetics, which disrupt Mcl-1 function with higher affinity and overcome resistance to available Bcl-2 family inhibitors [30, 50]. To date there are no viable clinical candidates. Here we evaluated the novel hydroxychinoline-derived small molecule inhibitor EU-5346 for its activity in Her2-positive BC cells under hypoxic conditions. Our data demonstrate EU-5346-induced cell death of Her2-positive BC cells and that Mcl-1 is required for cell killing. In addition we have found that, functionally, EU-5346 triggers ubiquitination of Mcl-1-bound Her2, indicating a previously unknown role for Mcl-1 to stabilize Her2 protein levels. Studies which delineate detailed molecular mechanisms by which Mcl-1 protects Her2 against ubiquitination and concomitant degradation are currently ongoing. In addition to Mcl-1/Her2-dependent survival pathways, our results indicate the existence of Mcl-1-dependent survival pathways in Her2-positive BC cells, which are independent of the Mcl-1–Her2 axis (Fig. 8). Importantly, siRNA-mediated knockdown of Mcl-1 and EU-5346 induced cell death also in BC cell lines resistant against Her2 inhibitors, trastuzumab and lapatinib in particular (Fig. 8); as well as in brain-primed Her2-positive BC cells resistant to Her2 inhibitors.

Mcl-1 mediates inherent resistance against widely used anticancer therapies including paclitaxel [26] and gemcitabine [27], as well as BH3 mimetics. Moreover, Mcl-1 may also contribute to the acquired resistance to specific BH-3 mimetics [51]. Silencing of Mcl-1 resensitized tumor cells to ABT-737 and decreased their resistance to anoikis [52, 53]. Owing to their functional redundancy, pan-active Bcl-2 inhibitors with at least some activity against Mcl-1 or the combination of Mcl-1 with other Bcl-2/Bcl-xL inhibitors are likely to achieve higher response rates than targeting one individual member of the Bcl-2 subfamily.

The use of multiprotein indexes has been proposed to predict response rates to single-agent Mcl-1 inhibitors or their combination with other inhibitors of the Bcl-2 family [54, 55]. However, in vivo studies are needed to confirm the utility of these indexes. Currently we are evaluating the in vitro anti-BC activity of EU-5346 in combination with ABT-199, and navitoclax, as well as with paclitaxel and gemcitabine.

Finally, our own and other data demonstrate high Mcl-1 protein levels also in Her2-negative BC cells, Luminal A-like BC and TNBCs in particular [25, 55–57]. Based on this protein profile, we evaluated whether *siMCL1* and EU-5346 induce cell death also in these BC subtypes. Indeed, our results demonstrate anti-BC activity in Luminal A-like (MCF-7) and TNBC (MDA MB-231) cells upon both siRNA-mediated downregulation of Mcl-1 (Additional file 1: Figure S3a,b) as well as treatment with EU-5346 (Additional file 1: Figure S3c,d), as evidenced by PARP cleavage (Additional file 1: Figure S3a,c), AlamarBlue® assays (Additional file 1: Figure S3b), and [^3^H]-thymidine uptake (Additional file 1: Figure S3d). Molecular mechanisms by which Mcl-1 induces cell death in these BC subtypes need to be further explored (Additional file 1: Figure S3e). Importantly, Balko et al. [58] recently demonstrated that TNBC cells residual after neoadjuvant chemotherapy express high levels of Mcl-1; and that Mcl-1 protects TNBCs from chemotherapy-induced apoptosis. These data support a therapeutic role of targeting Mcl-1 also in TNBC cells. Future investigations into whether a similar role for Mcl-1 also exists in other BC subtypes are of high interest.

## Conclusion

In summary, the present study adds another facet to the critical pathophysiologic role of Mcl-1 in BC pathogenesis in general, and Her2-positive BC cells in particular. The study thereby supports the clinical development of therapies, which target Mcl-1 alone or in combination with other therapies, both under normoxic and hypoxic conditions in order to further improve patient outcome in BC.

## Additional file

Additional file 1: Figure S1. Showing MCL-1 mRNA levels do not change in Her2-positive BC cells under hypoxic conditions. Figure S2 showing **a** Bcl-2 is expressed in both Mcl-1^wt/wt^ and Mcl-1^Δ/null^ MEFs. Whole-cell extracts were analyzed by immunoblotting with indicated antibodies. Immunoblotting for Erk2 confirmed equal protein loading. **b** EU-5346 does not change Mcl-1 mRNA expression in Her2-positive BC cells under hypoxic conditions. Figure S3 showing Mcl-1 is a potential therapeutic target not only in Her2-positive, but also in Luminal A-like and TNBC cells under hypoxic conditions. (PDF 1580 kb)

## Abbreviations

BC: Breast cancer
BH3: Bcl-2 homology 3
*CI*: Combination index
DKFZ: German Cancer Research Center
DMEM: Dulbecco’s modified Eagle’s medium
ER: Estrogen receptor
Erk2: Extracellular signal-regulated kinase 2
FACS: Fluorescence-activated cell sorting
FBS: Fetal bovine serum
FITC: Fluorescein isothiocyanate
Her2: Human epidermal growth factor receptor 2
Hif: Hypoxia-inducible factor
HR: Hormone receptor
Mcl-1: Myeloid cell leukemia-1
MEF: Murine embryonic fibroblast
MEM: Minimum essential medium
MTT: 3-(4,5-Dimethylthiazol-2-yl)-2,5-diphenyl tetrazolium bromide
PARP: Poly (ADP-ribose) polymerase
PEST: Proline, glutamic acid, serine, and threonine
PI: Propidium iodide
PR: Progesterone receptor
SD: Standard deviation
siRNA: Small interfering RNA
TNBC: Triple-negative breast cancer
