# Correction: Mcl-1 confers protection of Her2-positive breast cancer cells to hypoxia: therapeutic implications

**DOI:** 10.1186/s13058-024-01811-y

**Published:** 2024-04-02

**Authors:** Muhammad Hasan Bashari, Fengjuan Fan, Sonia Vallet, Martin Sattler, Melissa Arn, Claudia Luckner-Minden, Henning Schulze-Bergkamen, Inka Zörnig, Frederik Marme, Andreas Schneeweiss, Michael H. Cardone, Joseph T. Opferman, Dirk Jäger, Klaus Podar

**Affiliations:** 1 Department of Medical Oncology, National Center for Tumor Diseases (NCT), University of Heidelberg, Im Neuenheimer Feld #460, 69120 Heidelberg, Germany; 2 Department of Pharmacology and Therapy, Faculty of Medicine, Universitas Padjadjaran, Jl. Eijkman 38, Bandung, 02215 Indonesia; 3 Dana-Farber Cancer Institute, 450 Brookline Avenue, Boston, MA 02215 USA; 4 Eutropics, Inc., 767C Concord Avenue, Cambridge, MA 02138 USA; 5 German Cancer Research Center (DKFZ), Im Neuenheimer Feld 460, 69120 Heidelberg, Germany; 6 St. Jude Children’s Research Hospital, 262 Danny Thomas Place, Memphis, TN 38105 USA

**Correction: Breast Cancer Research (2016) 18:26** 10.1186/s13058-016-0686-4

Following the publication of the original article [1], the author reported that PARP blots of JIMT-1 and JIMT-1-BR3 cells in Fig. 7C of the original publication were mistakenly duplicated.

**Error image** 
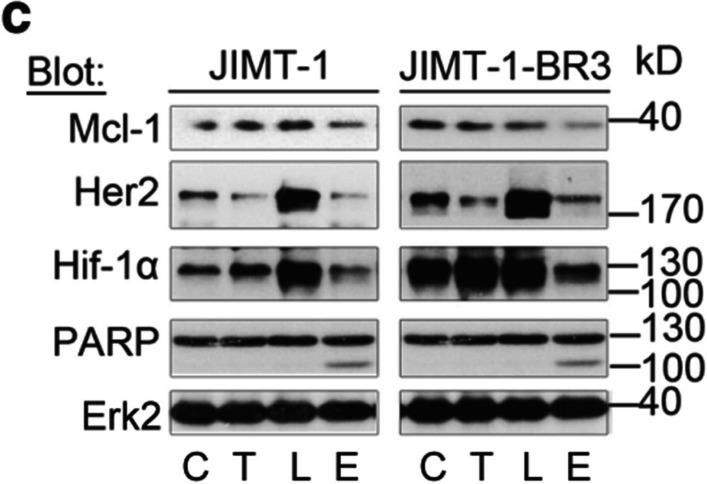


The correct figure below depicts the correct PARP blot for JIMT-1 cells.

**Correct image** 
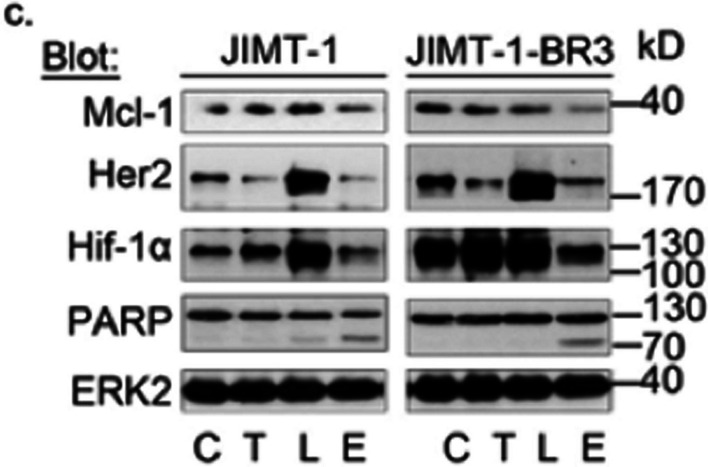


The error does not affect any of the interpretations or conclusions of the article.

The email address of the Corresponding Author has also been updated, from klaus.podar@nct-heidelberg.de to klaus.podar@krems.lknoe.at as shown in this Correction article.

The original article [1] has been updated.
