# Supplementary material for: Mcl-1 confers protection of Her2-positive breast cancer cells to hypoxia: therapeutic implications
Source: Breast Cancer Res. 2016 Feb 26;18:26. doi: 10.1186/s13058-016-0686-4 (PMC4769490; doi:10.1186/s13058-016-0686-4)
Supplement: Additional file 1: Figure S1. — Showing MCL-1 mRNA levels do not change in Her2-positive BC cells under hypoxic conditions. Figure S2 showing a Bcl-2 is expressed in both Mcl-1wt/wt and Mcl-1Δ/null MEFs. Whole-cell extracts were analyzed by immunoblotting with indicated antibodies. Immunoblotting for Erk2 confirmed equal protein loading. b EU-5346 does not change Mcl-1 mRNA expression in Her2-positive BC cells under hypoxic conditions. Figure S3 showing Mcl-1 is a potential therapeutic target not only in Her2-positive, but also in Luminal A-like and TNBC cells under hypoxic conditions. (PDF 1580 kb) [file 13058_2016_686_MOESM1_ESM.pdf]

Supplemental data 1

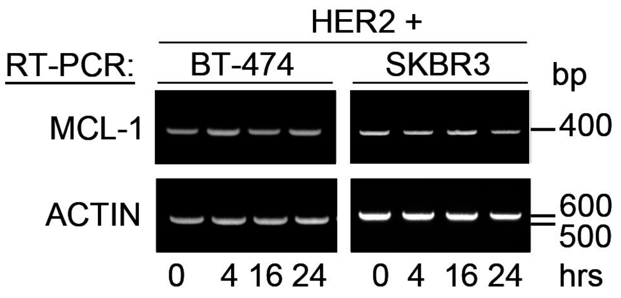

Supplemental data 2

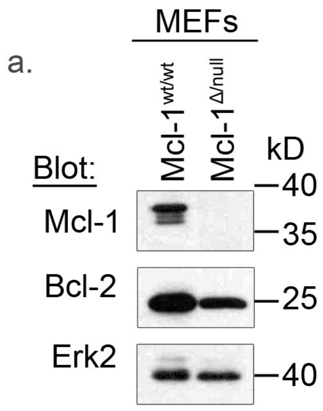

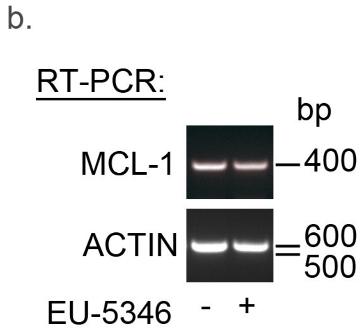

Supplemental data 3

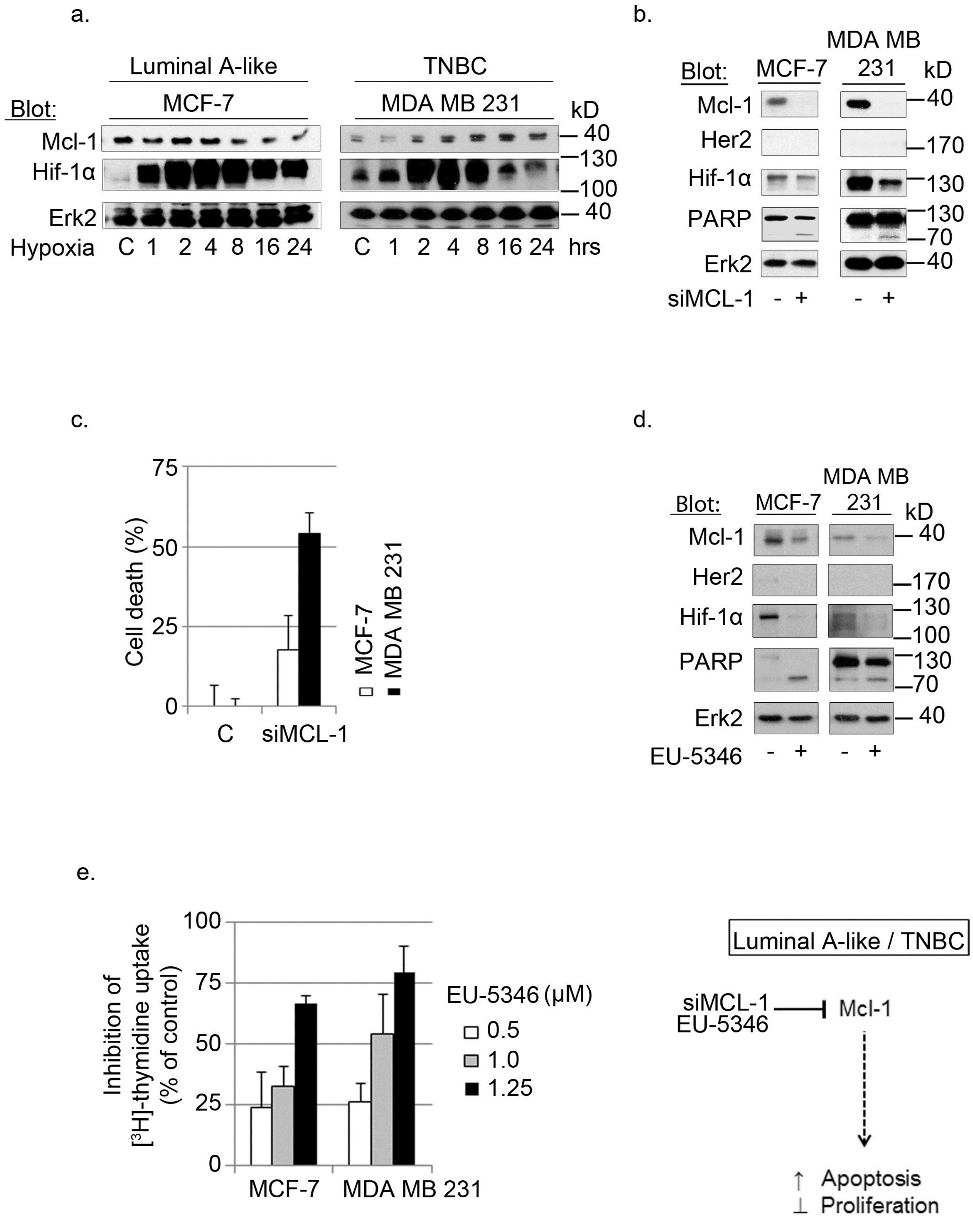

Supplemental data:

Supplemental data 1. MCL-1 mRNA levels do not change in Her2-positive BC cells under hypoxic conditions. Indicated BC cell lines were exposed to hypoxia for indicated time periods. Total RNA was purified from BC cells as described in *Materials and Methods* and analyzed by RT-PCR. β-ACTIN (ACTIN) served as a loading control. hrs, hours; bp, base pairs**;** C, normoxic control.

Supplemental data 2. (a) Bcl-2 is expressed in both Mcl-$1^{wt/wt}$ and Mcl-$1^{\Delta/null}$ MEFs. Whole-cell extracts were analyzed by immunoblotting with indicated antibodies. Immunoblotting for Erk2 confirmed equal protein loading. (b) EU-5346 does not change Mcl-1 mRNA expression in Her2-positive BC cells under hypoxic conditions. SKBR3 cells were treated with EU-5346 for 3 days then exposed to hypoxia for 6 hours. Total RNA was purified from BC cells as described in *Materials and Methods* and analyzed by RT-PCR. β-ACTIN (ACTIN) served as a loading control. hrs, hours; bp, base pairs.

Supplemental data 3. Mcl-1 is a potential therapeutic target not only in Her2-positive, but also in Luminal A-like and TNBC cells under hypoxic conditions. (a) Upregulation of Mcl-1 in TNBC cells but not in-Luminal A-like BC cells under hypoxic conditions. BC cell lines were incubated under hypoxic conditions for up to 24 hours. Whole-cell extracts were analyzed by immunoblotting with indicated antibodies; C, normoxic control (b,c). Genetic depletion of Mcl-1 induces apoptosis in both Luminal A-like (MCF-7) and TNBC cells (MDA MB-231). (b) BC cells were transfected with siMCL-1 for 2 days then exposed to hypoxia for 6 hours. Whole-cell extracts were analyzed by immunoblotting with indicated antibodies. Immunoblotting for Erk2 confirmed equal protein loading. (c) BC cells were transfected with siMCL-1 for 30 hours and then exposed to hypoxia for 2 days. Cell survival was determined by Alamar blue assay. Data represent mean ± *SD* for triplicate samples.

Results shown are representative of 3 independent experiments. (d,e) EU-5346 induces apoptosis in both Luminal A-like (MCF-7) and TNBC cells (MDA MB-231). BC cells were treated with EU-5346 for 3 days then exposed to hypoxia during the last 6 hours. Whole-cell extracts were analyzed by immunoblotting with indicated antibodies. Immunoblotting for Erk2 confirmed equal protein loading (d). BC cells were treated with EU-5346 for 3 days under hypoxic conditions. [^{3}H]-thymidine was added during the last 8 hours. Data represent mean ± *SD* for triplicate samples. Results shown are representative of 3 independent experiments. (e) Summary: Mcl-1 is a potential therapeutic target not only in Her2-positive, but also in Luminal A-like and TNBC cells under hypoxic conditions.
